# Normative video head impulse test data in subjects with and without vascular risk factors

**DOI:** 10.1007/s00405-020-06332-w

**Published:** 2020-09-10

**Authors:** Anders Hansson, Jonatan Salzer

**Affiliations:** 1 Department of Clinical Science, Neurosciences, Umeå University, Umeå, Sweden; 2 Department of Neurology, Umeå University, 90187 Umeå, Sweden

**Keywords:** Age effects, Neurotology, Vestibulo-ocular reflex (VOR), Video head impulse test (vhit), Normative data, Vascular disease

## Abstract

**Purpose:**

There is a paucity of age- and vascular risk factor-stratified video head impulse test (vHIT) vestibulo-ocular reflex (VOR) data in the literature. The aim of this study was to investigate the vHIT VOR properties in healthy subjects of different ages and subjects with vascular risk factors.

**Methods:**

This was a prospective observational single-center study at a tertiary referral university hospital in northern Sweden. Healthy participants and subjects with vascular risk factors were investigated with a floor standing external camera vHIT device. Age-stratified mean VOR gain among healthy adults and between group gain and gain asymmetry differences were calculated.

**Results:**

We included eighty-eight healthy adults with a mean (range) age of 50 (22–85) years and *n* = 48 stroke ward patients with vascular risk factors (but without vestibular disease) with a mean (range) age of 74 (42–92) years. The mean VOR gain of horizontal canals decreased at higher ages in healthy subjects (*r* = − 0.32, *p* < 0.01, *n* = 167 canals). The age-stratified mean (SD) VOR gains were < 30 years: 0.98 (0.07), 30–39 years: 0.97 (0.07), 40–49 years: 0.98 (0.06), 50–59 years: 0.99 (0.06), 60–69 years: 0.93 (0.08), ≥ 70 years: 0.89 (0.15). No consistent differences between healthy subjects and subjects with vascular risk factors were seen except for a trend towards more pronounced gain asymmetries in the latter group.

**Conclusions:**

Age, but not vascular risk factors influence VOR gain. Age-adjusted vHIT-measurements may be useful in acute vertigo stroke risk differentiation.

## Introduction

During acute vertigo with spontaneous or unilateral gaze-evoked nystagmus the head impulse test (HIT) may aid to differentiate between cerebrovascular disease and vestibular neuritis [1, 2]. During the bedside HIT the vestibulo-ocular reflex is tested by delivering fast unpredictable head rotations in the horizontal plane while the patient tries to maintain gaze on an earth fixed target. If catch-up saccades are noted by the examiner after the head rotation (overt saccades) the test is considered positive, i.e. indicating a peripheral lesion [3]. The video head-impulse test (vHIT) offers a quantitative and objective approach to detect VOR pathology which may also detect covert (i.e. during the head rotation) saccades [4–6]. By recording the eye and head velocities with high-speed cameras and computational software, saccades both overt and covert can be detected and the vestibulo-ocular gain can be calculated. Individual quantitative testing for all six semi-circular canals (SCCs) can be achieved by rotating the head in the plane of each canal [7, 8]. Two previous publications showed a decrease in VOR gain with increasing age [9, 10] while another one did not [11]. There is a paucity of published normative values for VOR gain and other vHIT measurements among subjects with vascular risk factors which are common in patients evaluated for suspected cerebrovascular disease.

This study aims to investigate the impact of vascular risk factors and age on the outcome of vHIT investigations, and to report normative values in healthy volunteers for all six SCCs using a floor standing external camera vHIT system from SYNAPSYS, Marseille, France.

## Materials and methods

### Subjects

All participants provided written informed consent. Subjects with vascular risk factors were recruited among patients treated for anterior circulation ischemic strokes or TIAs at the Stroke Ward at Umeå University Hospital during the summers of 2016 and 2017. Subjects were excluded for radiologically verified cerebellar or brainstem lesions, focal symptoms suggesting such lesions, a history of neurological or vestibular disease, hearing loss other than presbyacusis, current or chronic dizziness, recent (< 3 months) ophthalmic surgery, a history of head trauma or cervical spine injury or inability to provide informed consent, any prescribed drugs apart from those related to the vascular risk factors. Prior to vHIT subjects were interviewed about medical history and vascular risk factors and underwent a standard NIHSS assessment.

Healthy subjects were recruited mainly amongst hospital staff and relatives to inpatients and underwent vHIT as detailed below. The same exclusion criteria as above were applied together with any history of cerebrovascular disease or known vascular risk factors such as untreated hypertension, congestive heart disease, hyperlipidemia, diabetes, atrial fibrillation, ischemic heart disease, peripheral artery disease, lung embolism or deep vein thrombosis. Well-regulated hypertension was accepted in the older (≥ 70 years) age band.

### Cardiovascular risk stratification

To estimate cardiovascular risk load, the risk estimation model CHA_2_DS_2_-VASc was used. This risk estimation model is designed to predict ischemic stroke risk among subjects with atrial fibrillation [12]. The following risk factors for stroke were scored: Congestive heart failure (1), hypertension (1), age ≥ 75 years (2), diabetes mellitus (1), stroke/TIA or thromboembolism (2), vascular disease (1), age 65–74 years (1), sex category (one for female sex).

### Video head impulse test

Head impulses were recorded with the Ulmer Synapsys vHIT II system software version 14.1, as per the manufacturer’s instructions: At least five valid impulses per semicircular canal were collected. When testing the horizontal canals, the head was tilted forward at an angle of 30° to increase sensitivity [13]. Impulses were delivered in an unpredictable manner in time and direction in order to avoid voluntary head movements which may increase VOR gain [14]. The examiner’s hands were placed on the subjects forehead when applying rotation [15]. To maintain precision of VOR gain estimates, a horizontal canals variance threshold of ≤ 0.10 and a vertical canals variance threshold of ≤ 0.15 were applied as per the manufacturer’s instructions and we discarded visually identified outliers if needed to meet these criteria.

The VOR gain threshold values suggested by the manufacturer were ≥ 0.81 for the horizontal canals, and ≥ 0.71 for the vertical canals. Any canal with a mean gain lower than those thresholds was considered pathological. There is a paucity of normative threshold values for vHIT gain asymmetry.

### Statistical analyses and calculations

Descriptive statistics, analysis of correlations and calculations of gain asymmetry were made with IBM SPSS Statistics (release 24.0). The Shapiro–Wilks test was used to test for normality. Pearson’s Chi square test was used to compare proportions of canals with gain below cut off and saccades; the independent samples *t* test was used to compare gain means and the Mann–Whitney *U* test was used to compare gain asymmetry medians. Pearson’s correlation coefficient was used to test for correlations in continuous data and Spearman’s rho was used for non-parametric data.

Gain asymmetry (*G*_*A*_) was calculated in both horizontal and vertical planes as

$$ GA = \left[ {\frac{{\left( {G_{L} - G_{R} } \right)}}{{\left( {G_{L} + G_{R} } \right)}}} \right] \times 100, $$

where *G*_*R*_ denotes right sided mean gain and *G*_*L*_ denotes left sided mean gain [16]. Post-hoc power calculations suggested 97% power to detect a vHIT gain difference of 0.05 between the healthy subjects and those with vascular risk factors, SD 0.1, alpha 0.05. The study was approved by the regional ethical review board in Umeå (2014/284-31 with amendments) and conducted in accordance with the ethical standards of the Helsinki Declaration.

## Results

### Healthy subjects compared with subjects with vascular risk factors

Eighty-eight healthy subjects with a median age of 50 years and *n* = 48 subjects with vascular risk factors with a median age of 74 years participated in the study (Table 1). The group with vascular risk factors had mostly mild stroke symptoms with a median NIHSS score of 1 (range 0–8). Manual adjustments of insufficient pupil tracking were applied in *n* = 9 (10.2%) of healthy subjects and *n* = 8 (16.7%) subjects with vascular risk factors. Overall, the vHIT differences between the two groups were small without any consistent trends except for gain asymmetries which tended to be more pronounced in subjects with vascular risk factors (Table 2).

**Table 1:** Demographic properties of healthy subjects and subjects with vascular risk factors

	Healthy subjects, *n* = 88	Subjects with vascular risk factors, *n* = 48
Female, *n* (%)	61 (69)	20 (42)
Age, years, median (range)	50 (22–85)	74 (42–92)
Level of education^a^		
< 9 years	4 (5)	11 (23)
9 years	8 (9)	10 (21)
12 years	7 (8)	9 (19)
> 12 years	68 (77)	17 (35)
Risk factors		
Ever smoker	23 (26)	23 (48)
BMI, median (range)	23.9 (17.6–36.4)	27.4 (19.1–43.8)
Systolic blood pressure, median (range)	n/a	139 (112–189)
CHA_2_DS_2_VASc, median (range)	n/a	5 (2–8)
NIHSS, median (range)	n/a	1 (0–8)

*BMI* Body mass index’ *CHA*_*2*_*DS*_*2*_*VAS*c = Congestive heart failure, hypertension, age ≥ 75 years, diabetes mellitus, stroke/TIA or thromboembolism, vascular disease, age 65–74 years, sex category’ *NIHSS* national institute of health stroke scale. *n/a* not applicable

^a^Data on level of education missing in *n* = 1 healthy subject and *n* = 1 subject with vascular risk factors

**Table 2:** Vestibulo-ocular gain, gain asymmetry and saccades in all three semi-circular canal planes in healthy subjects and subjects with vascular risk factors

	Healthy subjects, *n* = 88	Subjects with vascular risk factors, *n* = 48	*p* value
Bilateral Horizontal			
Valid canals^a^	*n* = 167	*n* = 87	
Gain, mean (SD)	0.95 (0.09)	0.93 (0.11)	0.09
Median (range) gain asymmetry	1.5 (0–37.4)	2.3 (0–37.3)	0.19
* N* (%) canals with gain below cut-off^b^	10/167 (6.0)	10/87 (11.5)	0.12
* N* (%) canals with saccades	28/167 (16.8)	14/87 (16.1)	0.89
Anterior canals			
Valid canals^c^	*n* = 168	*n* = 83	
Gain, mean (SD)	0.95 (0.14)	0.99 (0.11)	0.03
* N* (%) canals with gain below cut-off^d^	10/168 (6.0)	1/83 (1.2)	0.08
* N* (%) canals with saccades	29/168 (17.3)	12/83 (14.5)	0.57
Posterior canals			
Valid canals^c^	*n* = 170	*n* = 84	
Gain, mean (SD)	0.84 (0.15)	0.80 (0.14)	0.02
* N* (%) canals with gain below cut-off^d^	23/170 (13.5)	16/84 (19.0)	0.25
* N* (%) canals with saccades	34/170 (20.0)	21/84 (25.0)	0.36
LARP			
Median (range) gain asymmetry	5.4 (0–91.8)	10.2 (1.5–27.0)	< 0.01
RALP			
Median (range) gain asymmetry	5.7 (0–138.5)	9.5 (0–52.5)	0.13

The functional LARP and RALP planes were used for gain asymmetry calculations rather than bilateral anterior and posterior. The independent *t* test was used to compare means, the Mann–Whitney *U* test to compare medians, and the Chi square test to compare proportions

*SD* Standard Deviation, *LARP* Left Anterior, *Right* Posterior, *RALP* Right Anterior, *Left* Posterior

^a^A valid horizontal canal had a sigma ≤ 0.1, and at least *n* = 5 approved impulses

^b^manufacturer specified pathological gain cut off in horizontal canals < 0.81

^c^A valid vertical canal had a sigma ≤ 0.15, and at least *n* = 5 approved impulses

^d^manufacturer specified pathological gain cut off in vertical canals < 0.71

### Vestibulo-ocular reflex gain by age and cardiovascular risk factor load

The horizontal, anterior and posterior canals mean gains decreased with increasing age in healthy subjects (*r*_horizontal_ = − 0.32, *p* < 0.01, *n* = 167 canals; *r*_anterior_ = − 0.23, *p* < 0.01, *n* = 168 canals; *r*_posterior_ =  − 0.58, *p* < 0.01, *n* = 170 canals), absolute means values displayed in Table 3. In subjects with vascular risk factors this association was only seen in posterior canals (*r*_horizontal_ = − 0.15, *p* = 0.18, *n* = 87 canals; *r*_anterior_ = 0.05, *p* = 0.63, *n* = 83 canals; *r*_posterior_ = − 0.31, *p* < 0.01, *n* = 84 canals). There was no correlation between horizontal or anterior canal mean gain and risk factor load (CHA_2_DS_2_-VASc) in subjects with vascular risk factors (*r*_horizontal_ = 0.02, *p* = 0.85, *n* = 87 canals; *r*_anterior_ = 0.10, *p* = 0.35, *n* = 83 canals), however again, such a correlation was seen for posterior canals (*r*_posterior_ =  − 0.23, *p* = 0.04, *n* = 84 canals).

**Table 3:** Horizontal, anterior and posterior canal mean (SD) vestibulo-ocular reflex gain measured with the video head impulse test by age strata in *n* = 88 healthy subjects

Age	Mean (SD) gain	Number of canals
*Horizontal canals*		
< 30	0.98 (0.07)	29
30–39	0.97 (0.07)	30
40–49	0.98 (0.06)	26
50–59	0.99 (0.06)	20
60–69	0.93 (0.08)	33
≥ 70	0.89 (0.15)	29
*Anterior canals*		
< 30	0.95 (0.09)	31
30–39	0.98 (0.08)	30
40–49	1.00 (0.07)	25
50–59	0.96 (0.10)	20
60–69	0.93 (0.15)	35
≥ 70	0.85 (0.25)	27
*Posterior canals*		
< 30	0.92 (0.08)	30
30–39	0.93 (0.07)	30
40–49	0.90 (0.07)	28
50–59	0.88 (0.10)	20
60–69	0.79 (0.09)	34
≥ 70	0.66 (0.21)	28

*SD* Standard deviation

## Discussion

This study investigated vestibulo-ocular reflex data from video head impulse tests among healthy subjects and subjects with vascular risk factors but without vestibular diseases or posterior fossa stroke. The vestibulo-ocular gain decreased slightly with increasing age among healthy subjects but few consistent differences between healthy subjects and subjects with vascular risk factors were found. This suggests that there is little need for concern that vascular risk factors may spoil the video head impulse test measurements, thus this test paradigm is probably a valid tool to identify damage in the vestibular system during acute onset vertigo work-up among patients at risk for stroke. However, to avoid false positive findings, video head impulse test devices would probably need age adjusted gain cut-offs which to the knowledge of the authors has not yet been adopted.

The lack of correlation between vascular risk factors and horizontal canal vestibulo-ocular reflex gain among subjects with vascular risk factors suggests that it is not the risk factor load per se that drives the age-dependent decline in vestibulo-ocular reflex gain seen by us and others, [17–19] but rather some other age-related change(s) in the tissues and functions of the vestibulo-ocular reflex. Subjects recovering from stroke have been shown to exhibit post-stroke fatigue leading to reduced attention [20], which may explain the trend towards greater gain asymmetries in subjects with vascular risk factors.

Some vestibulo-ocular reflex gains were > 1. This could be due to software gain calculation characteristics or miscalibration of the measurement system. Whether this “overshoot” is consistent over the gain range, or only attributed to the impulses of the highest gain remains to be investigated, as consistently miscalculated gain values may lead to false negative tests.

The healthy subjects were younger than the group with vascular risk factors and this may have interfered with the analyses, although it should be noted that despite this baseline skewness mean horizontal gain values did not differ between the groups. The mean anterior and posterior gains differed slightly (0.04) between the groups; this was interpreted as without clinical significance. The age-related gain decline seen in healthy subjects was evident only at higher ages (≥ 60 years) which may explain the relative lack of such a trend among the subjects with vascular risk factors with a median age of 74 years. A weakness of this study is the lack of repeated measurements (preventing investigation of the test–retest reliability) and also the lack of parallel testing with different video head impulse test devices which may have been informative to highlight device-dependent differences regarding the findings.

In summary this study suggests that age, but not vascular risk factors, influence vestibulo-ocular reflex gain measured with the video head impulse test. Future studies in this area should focus on using the video head impulse test in acute vertigo stroke risk differentiation, potentially also investigate how applying age-dependent cut-offs for vestibulo-ocular reflex gain influence the results.
